# Students’ perception about mental illness

**DOI:** 10.4103/0972-6748.62267

**Published:** 2009

**Authors:** R. K. Mahto, P. K. Verma, A. N. Verma, A. R. Singh, S. Chaudhury, K. Shantna

**Affiliations:** Department of Psychiatric Social Work, RINPAS, Kanke, Ranchi; 1 Department of Psychiatric Social Worker, RINPAS, Kanke, Ranchi; 2 Department of Clinical Psychology, RINPAS, Kanke, Ranchi; 3 Department of Psychiatry, RINPAS, Kanke, Ranchi

**Keywords:** Mental illness, Opinion, Students

## Abstract

**Background:**

In developing countries like India, there are evidences that stigma associated with mental illness is increasing. As in parts of the developing world, with advancement of urbanization and rapid industrialization, people tend to react in a very peculiar and biased way when they confront a mentally ill person.

**Materials and Methods:**

The present study aimed to find out students’ opinion about mental illness. A total of 100 students (50 male and 50 female) from Ranchi University were purposively recruited for the study, and the 51-item Opinion about Mental Illness (OMI) Scale was administered.

**Results:**

Majority of the students were from Hindu families, of whom 42 (84%) were males and 38 (68%) were females. With regard to OMI scale, the item, viz., ‘The law should allow a woman to divorce her husband as soon as he has been confined in mental hospital with a severe mental illness’, both male (46%) and female (56%) students were neutral (significant at 0.014, *P* < 0.05).

**Conclusion:**

Overall no significant level of difference emerged between male and female students with regard to opinion about mental illness.

All over the world, there is an increasing awareness of mental illness as a significant cause of morbidity. This awareness has increased with the steady decline of morbidity due to nutritional disorders, communicable diseases and other forms of physical illness, especially in countries undergoing epidemiological transition. Mental and behavioral disorders are common, affecting more than 25% of all people at some time during their lives. The point prevalence of mental illness in the adult population at any given time is about 10%. Similarly, around 20% of all patients seen by primary health care providers have one or more mental health disorders (Kabir *et al*., 2004). Opinion about mental illness plays vital role in long-term care of mentally ill patients. Not only people but patients who are mentally ill also frame a different picture about their illness which can be neutralizing by public’s familiarity with serious mental illness, which subsequently will decrease stigma (Patrick *et al*., 2001). People frame a picture about mental illness and mentally ill patients in their mind, which generally guides their behavior, so public must be educated to bring about positive changes in attitude (Song *et al*., 2005). It has always been an important area of investigation among mental health professionals. Every section of society has its unique way of perception about mental illness, particularly the young generation and college-going students. College has remained the best place to develop a comprehensive mental health program, because the attitude and values of college-going students influence the society most. Research has convincingly shown that people who have experienced psychiatric problems are feared, disliked and broadly rejected by society, and in the aggregate it makes the presence of stigmatization plain. Additionally, recent studies have shown that stigmatizing attitudes towards people with mental illness are widespread (Byrene, 1997; Link *et al*., 1997; Jorm *et al*., 1999) and are commonly held (Porter, 1998). People tend to react in a very discriminating way towards mentally ill patients almost in every section of society (Farina, 1998). People with severe mental illness are never viewed more favorably than someone with any other defaming disadvantages (e.g., blindness, cancer, dwarfism, leprosy, etc.). Rather, typically those with mental illness are regarded as abhorrent or social pariahs (Farina, 1998). An insane person is regarded as more dangerous, bad and foolish than a ‘leper.’ Neki (1966) also shared the same finding in his study that was carried out as a research project at Amritsar, which was funded by the Indian Council of Medical Research (ICMR). On the basis of a survey, he drew the conclusion that a sizable section of population fears and tends to strangely reject the mentally ill patients. Moreover, lack of scientific knowledge about mental illness further encourages this kind of behavior (Wolff *et al*., 1996). On the other hand, cross-sectional studies have shown that members of the general public who have more knowledge about mental illness are less likely to endorse stigmatizing attitudes (Link *et al*., 1997).

Indian studies indicate that the public, including the educated urban groups, is largely misinformed about the various aspects mental health, and information possessed by them remains uncrystallized. The mentally ill are perceived as aggressive, violent and dangerous and not fit for coexistence in society. A tendency to maintain social distance from mentally ill and to reject them socially still persists and makes its existence felt. Studies addressing this issue have pointed out that a great deal of misconceptions, superstitions and ignorance with regard to mental illness exists in our society, which makes the picture more distorted.

## MATERIALS AND METHODS

The study was done in the PG departments of Ranchi University and St Xavier’s College, Ranchi. A total of 100 students (50 males and 50 females) were purposively recruited for the present study. The sample comprised of both males and femalesin the age range of25-35 yearsand educated minimum up to the graduate level. Furtherstudents having major psychiatric or physical illnessand with family history of mental illness were excluded.

### Tools administered

#### Socio-demographic data sheet specially designed for the present study

It was applied to elicit socio-demographic details of the subjects.

Opinion about mental illness scale (Cohen and Struening, 1962). It was applied to elicit opinion of the students about mental illness. It contains 51 items that measure attitude towards, and causes and treatment of, mental illness. There are 5 factors: (A) authoritarianism, (B) benevolence, (C) mental hygiene, (D) Social restrictiveness and (E) interpersonal etiology. Individual responses to each item had 6 alternatives, viz., strongly agree, agree, not sure but probably agree, not sure but probably disagree, disagree, and strongly disagree. Each factor or dimension in the test was defined by a particular group of items. Responses to these items were rated from 1 (strongly agree) to 6 (strongly disagree) regardless of the direction (positive or negative) of the items. In computing the factor score, each item received the appropriate no according to the respondents’ position in agree-disagree continuum.

### Statistical analysis

It was done using appropriate statistics, i.e., percentage; and to find out significant differences between male and female groups, χ^2^ was used. SPSS version 13.0 was applied for the analysis of raw data.

## RESULTS

Table 1 shows that majority of the subjects belonged to a Hindu family. Most of them were unmarried. Majority of the subjects were from rural areas and had average socioeconomic status.

**Table 1:** Socio-demographic characteristics of the population

Variables	Male *n* = 50 (%)	Female *n* = 50 (%)
Religion		
Hindu	42 (84)	38 (68)
Muslim	4 (8)	7 (14)
Christian	3 (6)	5 (10)
Others	1 (20)	0 (0)
Domicile		
Urban	15 (30)	9 (18)
Rural	35 (70)	41 (82)
Marital status		
Married	0 (0)	1 (2)
Unmarried	50 (100)	49 (98)
Socioeconomic status		
Upper	07 (14)	02 (4)
Middle	38 (76)	40 (80)
Lower	05 (10)	08 (16)

## DISCUSSION

Majority of the subjects belonged to a Hindu family, viz., 84% from among males and 68% from among females. Most of them were unmarried, viz., 50% among males and 49% among females, respectively. Many of them were from rural areas, viz., 70% among males and 82% among females; and had average socioeconomic status, viz., 76% and 80% of male and female students, respectively, which simply denotes predominately Hindu populated areas. Middle class families widely represented the sample. Most of the subjects were unmarried, which indicates that students were career oriented.

A review of 27 studies found that 14 studies revealed no significant difference between male and female subjects’ opinion on mental illness (Farina, 1998). People have tendency to avoid such issues, which is also reflected in Table 2, item no. 7, where majority of female students (52%) were neutral when they were asked about their opinion that state people who are mentally ill let their emotion control them. This was found significant at (0.020, *P* < 0.05) level. This was also found in the study by Farina (1998), in which she stated that male and female students were reluctant to come out with any definite response when probed in these areas. From this, it can be concluded that people tend to avoid such questions and are very uncomfortable with any comments due to lack of knowledge. Male and female students’ opinions with regard to patients’ marital life were obviously different, where male students (46%) were more neutral when they were asked about their opinion on item no. 37 of OMI scale, which is about the law that should allow a women to divorce her husband as soon as he becomes confined to a mental hospital with a severe mental illness. Here male respondents showed more tolerance in comparison to female respondents. Majority of the students (female, 56%; male, 46%) also had a neutral attitude when they were asked about the same (significant at 0.014, *P* < 0.5 level). This could be explained on the basis of the understanding that female students are more concerned about their future generation, about their life partner and tend to get more sophisticated life than men. In general, Dovido *et al*. (1985) concluded that people are ambivalent in their attitude towards persons with psychological problems and deliberately ignore the issue. In the present study too, majority of the females demonstrated ambivalent attitude when they were asked about sensitive issues related to persons with major psychological problems. Interestingly, majority of the female students came up with more neutral responses with regard to their opinions about mental illness, as indicated in Table 2, which points towards the general tendency of people to avoid such issues as far as possible, and this was more apparent in the students’ groups in this study.

**Table 2:** Answers of subjects on ‘opinion about mental illness scale items’

Male students			Female students			χ^2^ (df)	Significance
Agree	Neutral	Disagree	Agree	Neutral	Disagree		
32	34	34	20	46	34	2.285(2)	0.319
42	12	46	58	10	32	2.627	0.269
64	24	12	68	22	10	0.195	0.907
16	22	62	4	28	68	4.098	0.129
62	24	14	56	20	24	1.650	0.438
38	26	36	30	52	18	1.564	0.457
38	26	36	30	52	18	7.804*	0.020 *P* < 0. 05
46	36	18	48	32	20	0.192	0.909
26	8	6	70	20	10	3.892	0.143 NS
52	28	20	52	34	14	0.820	0.664 NS
54	22	24	50	42	8	7.202	0.027
80	16	4	92	6	2	3.025	0.220 NS
34	50	16	38	40	22	1.140	0.565
40	28	32	36	26	38	0.399	0.819
24	36	40	34	30	36	1.240	0.538
46	32	22	52	36	12	1.772	0.412
80	16	4	60	34	6	4.869	0.088
74	18	8	76	22	2	2.013	0.365
78	18	4	68	22	10	1.828	0.401
36	48	16	36	52	12	0.366	0.833
76	20	4	68	16	16	4.044	0.132
80	14	6	80	12	18	0.220	0.896
68	18	14	20	14	6	2.336	0.311
22	32	46	10	48	42	3.941	0.139
58	34	8	64	26	10	0.792	0.673
20	48	32	12	54	34	1.207	0.547
72	20	8	64	24	12	0.817	0.665
64	30	6	52	36	12	1.893	0.388
22	22	56	12	18	70	2.448	0.294
30	48	22	24	62	16	1.474	0.479
18	12	70	10	16	14	1.484	0.476
16	16	68	6	36	58	6.516	0.038
32	40	28	28	44	28	0.229	0.892
14	30	56	10	26	64	0.743	0.690
16	30	54	14	30	56	0.085	0.958
62	26	12	62	22	16	0.452	0.798
26	46	28	24	56	20	8.589*	0.014 *P* < 0. 05
62	26	12	48	40	12	0.2376	0.305
54	26	20	58	32	10	2.048	0.359
18	28	54	12	30	58	0.706	0.703
26	46	28	24	56	20	1.197	0.550
74	16	10	80	14	6	0.684	0.711
14	24	62	26	56	18	20.300*	0.000 *P* < 0. 05
30	44	26	10	54	36	6.317*	0.042 *P* < 0. 05
50	38	12	66	22	12	3.237	0.198
20	30	50	30	32	38	1.805	0.396
82	12	6	20	12	8	0.155	0.925
78	18	4	74	22	4	0.253	0.881
54	24	22	28	36	36	7.012	0.030
40	30	30	36	34	30	0.230	0.891
30	50	20	26	40	34	2.513	0.285

Item numbers 43 and 44 of OMI scale, mentioned in Table 2, signify that college professors are more likely to become mentally ill than are businessmen, and many people who have never been patients in a mental hospital are more mentally ill than hospitalized mentally ill patients, respectively. It was found that 62% of male students disagreed with, whereas 56% of female students had neutral attitude towards, the statement that college professors are more likely to become mentally ill than are businessmen. It was found that male students had clear-cut opinion whereas female students tended to avoid such questions and gave neutral responses (found significant at 0.000, *P* < 0.05 level).

Similarly 44% of male students and 54% of female students had neutral attitude towards OMI scale items that stated ‘many people who have never been patients in a mental hospital are more mentally ill than many hospitalized mentally ill patients (significant at 0.042, *P* < 0.05 level). Among male respondents, 30% were aggraded that people who had never been in a mental hospital are more mentally ill than the hospitalized patients. This may be due to lack of awareness about mental illness among general public. Farina (1973 and 1981) found that the public tends to be more tolerant of deviant conduct when it is not described using mental illness labels. In the present study too, many respondents seemed ignorant about scientific facts related to mental illness and mentally ill persons, because most of them were in the 18-35 years age group and were engaged in their studies. They might not have had enough exposure to this field; that is why probably they all seemed ignorant even though hailing from Ranchi, where a very famous mental hospital is located. In the study by Pandey *et al*. (2008), it was found that psychiatric ward attendants had more positive attitudes than the general attendants towards psychiatric illnesses. Socio-demographic variables like older age, higher education and longer duration of contact with the psychiatrically ill predicted more favorable attitudes. Findings of the present study can be justified on the basis that no respondent had any exposure to psychiatric field earlier. Less exposure to psychiatric field adversely determines attitude towards mental illness. Similarly in item no. 49 of OMI scale, which signifies that there is little that can be done for the mentally ill patients in mental hospitals except see that they are comfortable and well fed, 54% of the male respondents were in agreement with this statement and expressed the view that mental hospital has nothing extra to do with such mental patients except conventional services that are being delivered by the hospital to these patients. This reveals the narrow-mindedness of the general public. However, no significant finding could emerge with regard to this statement.

The general trend of the studies carried out in India so far indicates mixed results and highlights the fact that lay urban public is largely misinformed about the various aspects of mental health, and the information possessed by it remains uncrystallized. The present study likewise shows the same picture, wherein female students displayed neutral or ambivalent attitude; however, male students were found to have very sharp opinions about some aspects, viz., OMI scale item numbers 3, 5, 12, 47 and 48, wherein male students strongly agreed with the statements, viz., ‘Most patients in a mental hospital are not dangerous,’ ‘If patients love their children more, there would be less mental illness,’ ‘Even though patients in mental hospitals behave in funny ways, it is wrong to laugh about them,’ ‘Our mental hospitals should be organized in a way that makes the patients feel as much as possible like they are living at home,’ and ‘One of the main causes of mental illness is lack of moral strength or willpower,’ respectively. This shows the optimistic and sympathetic attitude of male students. Exposure of male students to the outer world, which subsequently shapes their perception, could be a possible reason for male students coming out with such responses; however, no significant findings could emerge.

### Limitations of the study

Small sample size, viz., 100 participants, is definitely not good enough to make conclusive remarks with regard to ‘opinion about metal illness’. The same study can be replicated with a larger sample size and by including larger geographical areas. Considering the above limitations, findings of the present study should be generalized cautiously.

## CONCLUSION

Overall no significant difference emerged between male and female students with regard to opinion about metal illness.

## References

[CIT1] Byrne P (1997). Psychiatric Stigma, Past, Passing and to come. J Royal Society Med.

[CIT2] Corrigan P. W, Green A, Lundin R, Kubiak M. A, Penn D. L (2001). Familiarity with and social distance from people who have serious mental illness. Psychiatr Serv.

[CIT3] Jorm A. F, Wright A. M (2008). Influences on young people’s stigmatizing attitudes towards peers with mental disorders: National survey of young Australians and their parents. The British Journal of Psychiatry.

[CIT4] Jorm A. F, Jacobsn P. A, Chrisztensen H (1999). Attitude towards people with mental disorder: A survey of the Australian Public and health professionals. Aust NZ J Psychiatry.

[CIT5] Kabir M, Zubair I, Isa S, Abubakar, Aliyu M. H (2004). Perception and beliefs about mental illness among adults in Karfi village, northern Nigeria. BMC International Health and Human Rights.

[CIT6] Lauber C, Nordt C, Haker H, Falcato L, Rössler W (2006). Community Psychiatry: Results of a Public Opinion Survey, International Journal of Social Psychiatry.

[CIT7] Link B, Strueming E. L, Rahav M, Phelan J. C, Nattlerock L (1997). on stigma and its cosequences: Evidence from a longitudinal study of men with dual diagnosis of mental illness and substance abuse. J Health Soc Behavi.

[CIT8] Pandey V, Saddichha S, Kumar R (2008). Attitudes of Ward Attendants towards Mental Illness: Comparisons and Predictors. International Journal of Social Psychiatry.

[CIT9] Song L.Y, Chang L.Y, Shih C. Y, Lin C. Y, Yang M.J (2005). Community Attitudes Towards the Mentally Ill: The Results of a National Survey of the Taiwanese Population. International Journal of Social Psychiatry.

[CIT10] Porter R (1998). Can the stigma of mental illness be changed?. Lancet.

[CIT11] Farina A, Muesser K. T, Tarrier N (1998). Stigma. Handbook of social functioning in schizophrenia.

[CIT12] Neki J. S (1966). Problems of motivation affecting the psychiatrists, the general practitioner and the public in their interaction in the field of mental health. Indian Journal of Psychiatry.

[CIT13] Wolf G, Pathare S, Carig T, Leff J (1966). Community attitudes to mental illness. Britsh Journal of Psychiatry.

[CIT14] Dovido J. F, Fishbane R, Sibicky M (1985). Perception of people with Psychological problems: Effects of seeking counseling. Psychol Rep.

